# Adjuvant chemotherapy can benefit the survival of stage I lung adenocarcinoma patients with tumour spread through air spaces after resection: Propensity-score matched analysis

**DOI:** 10.3389/fonc.2022.905958

**Published:** 2022-08-16

**Authors:** Shaonan Xie, Qingyi Liu, Yaqing Han, Shize Wang, Huiyan Deng, Guangjie Liu

**Affiliations:** ^1^ Department of Thoracic Surgery, The Fourth Hospital of Hebei Medical University, Shijiazhuang, China; ^2^ Department of Pathology, The Fourth Hospital of Hebei Medical University, Shijiazhuang, China

**Keywords:** lung adenocarcinoma, stage I, spread through air spaces(STAS), adjuvant chemotherapy, prognosis

## Abstract

**Background:**

It is still unclear whether stage I lung adenocarcinoma patients with tumour spread through air spaces (STAS) can benefit from postoperative adjuvant chemotherapy (ACT) after lobectomy. This study investigated the effect of ACT on the postoperative survival of patients with stage I (STAS^+^) lung adenocarcinoma.

**Methods:**

We retrospectively analysed the clinical data of stage I (STAS^+^) invasive lung adenocarcinoma patients who underwent lobectomy in the Department of Thoracic Surgery of our hospital from January 1, 2013 to January 1, 2016. Propensity score matching (PSM) was performed to group patients to investigate whether ACT could lead to better prognosis of patients.

**Results:**

A total of 593 patients with stage I (STAS^+^) lung adenocarcinoma were enrolled. The study after PSM included 406 patients. Kaplan–Meier survival analysis showed the experimental group had a better 3-year recurrence-free survival (RFS) rate (*p* = 0.037) and the 5-year RFS rate (*p* = 0.022) than the control group. It also had higher 5-year overall survival (*p* = 0.017). The multivariate analysis by Cox proportional hazard regression model showed that stage I STAS^+^ lung adenocarcinoma patients with lymphatic vessel invasion (HR: 1.711, 95% CI: 1.052-2.784; *p* = 0.045), vascular invasion (HR: 5.014, 95% CI: 3.154-7.969; *p* < 0.001), and visceral pleural invasion (HR: 2.086, 95% CI: 1.162-3.743; *p* = 0.014), and without ACT (HR: 1.675, 95% CI: 1.043-2.689; *p* = 0.033) had a significant survival disadvantage.

**Conclusion:**

ACT can boost the postoperative survival of patients with stage I (STAS^+^) lung adenocarcinoma.

## Introduction

Lung cancer is still the leading cause of death of cancer patients worldwide (1). Surgical resection is still the preferred treatment for patients with stage I, stage II, and in some cases stage III non-small-cell lung cancer (NSCLC). For stage II and III patients, postoperative adjuvant chemotherapy (ACT) can improve their survival (2), but for stage I patients, whether survival is boosted by ACT is still inconclusive. The National Comprehensive Cancer Network (NCCN) recommends that ACT be given to stage I patients with high-risk factors, including tumours ≥ 4 cm, lymphatic vascular invasion, visceral pleural invasion, poorly differentiated cancer, incomplete lymph node sampling, and wedge resection (3).

Lung adenocarcinoma is the most common histological type of NSCLC, accounting for approximately 50% of all NSCLC (4). In 2011, the International Association for the Study of Lung Cancer, American Thoracic Society and European Respiratory Society (IASLC/ATS/ERS) classified adenocarcinomas into lepidic, acinar, papillary, micropapillary, solid, and mucinous types based on the growth pattern of invasive lung adenocarcinoma (5). Micropapillary and solid lung adenocarcinoma have worse prognoses than the lepidic, acinar, and papillary types. In addition, this stage IB micropapillary/solid invasive adenocarcinoma obtains survival benefit from ACT (6, 7). In recent years, tumour spread through air spaces (STAS) has come to be considered a new invasion method for stage I invasive lung adenocarcinoma. STAS has been associated with a high recurrence and metastasis rate and a poor survival rate (8–10). There is growing evidence of a correlation between the surgical resection method and the survival rate of STAS-positive lung adenocarcinoma patients (11, 12). Lobectomy has a better prognosis than segmentectomy (12, 13). In our clinical work, we have observed that STAS^+^ stage IA2-IA3 patients have worse survival than STAS^–^ patients, which has been confirmed by others (14). There is no consensus on whether patients with stage I (STAS^+^) lung adenocarcinoma who undergo lobectomy, especially stage IA lung adenocarcinoma, can benefit from ACT.

In this study, we retrospectively analysed the clinical data of patients with stage I (STAS^+^) who underwent lobectomy. We grouped all patients according to propensity score matching (PSM) to study the survival benefit of ACT after lobectomy in patients with stage I (STAS+) lung adenocarcinoma.

## Materials and methods

### Patient selection

**Figure 1:**
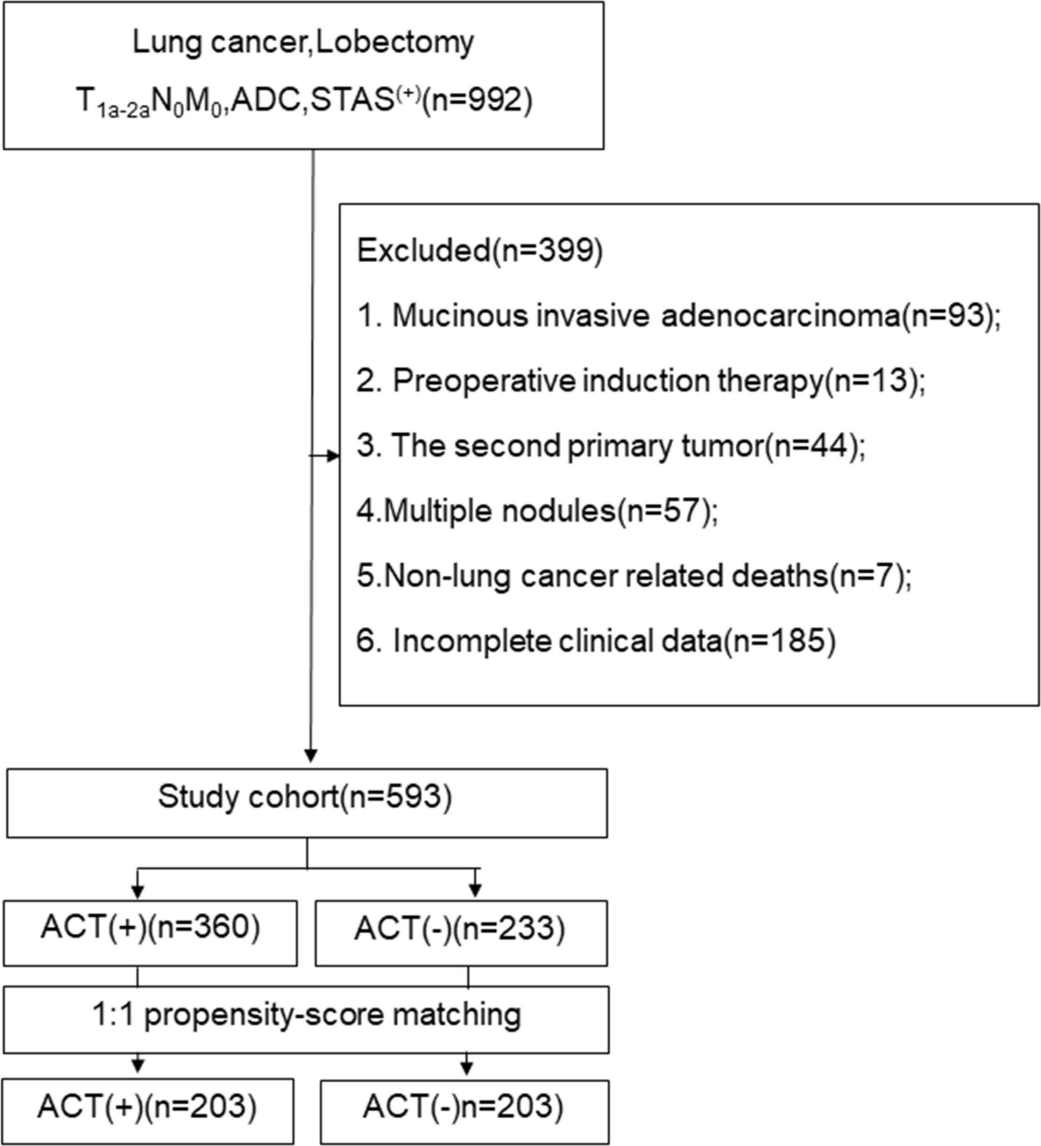
The follow chart. ADC, adenocarcinoma; STAS, spread through air space; ACT, adjuvat chemotherapy.

The medical ethics committee of the Fourth Hospital of Hebei Medical University, China, approved this study (ethics number:2021ky103), and the ethics committee waived the requirement of informed consent from patients. We retrospectively analysed the clinical data of stage I (STAS^+^) invasive lung adenocarcinoma patients who underwent lobectomy and systematic nodal dissection in the Department of Thoracic Surgery of our hospital from January 1, 2013 to January 1, 2016. Routine preoperative examination was performed to exclude metastasis. Patients who met the following conditions were excluded from the study: 1. mucinous invasive adenocarcinoma; 2. preoperative induction therapy; 3. the second primary tumor; 4. Multiple nodules; 5. Non-lung cancer related deaths;and 6. Incomplete clinical data. The flow chat show in **Figure 1**.

### Histopathological evaluation of STAS

**Figure 2:**
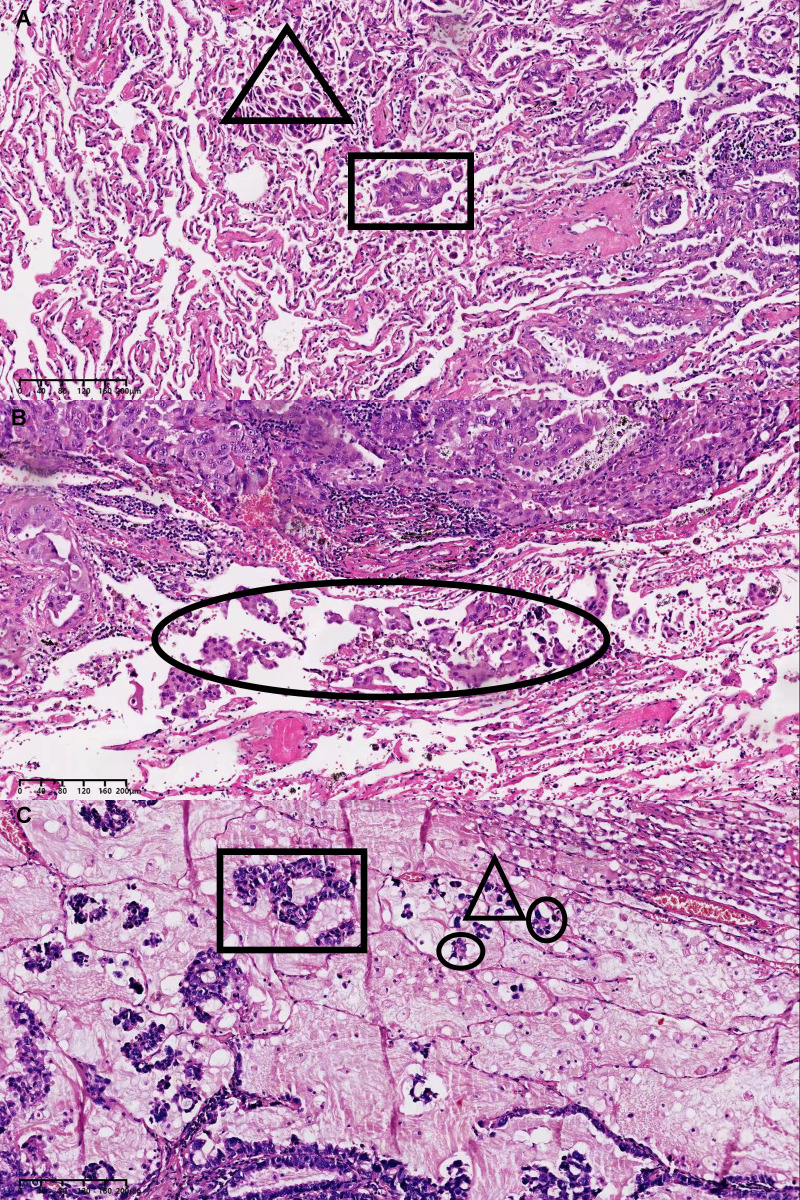
**(A)** STAS manifest morphologically as scattered single cancer cells in the triangular area and solid nests in the rectangular area. **(B)** STAS manifest morphologically as micropapillary clusters in the elliptical area. **(C)** STAS manifest morphologically as scattered single cancer cells in the triangular area, micropapillary clusters in the elliptical area and solid nests in the rectangular area. STAS, spread through air space.

All specimens were immediately fixed in formalin after surgical resection and stained with haematoxylin and eosin. The sections were independently evaluated by two experts in the Department of Pathology, and the specimens were classified according to the IASLC/ATS/ERS histological classification of lung invasive adenocarcinoma (5). The growth pattern that accounted for the largest proportion (even if <50%) was used to classify the lung invasive adenocarcinoma into the lepidic, acinar, micropapillary, papillary, or solid type. STAS lesions are composed of tumour cells, which manifest morphologically as scattered single cancer cells, micropapillary clusters, and solid nests located in the normal alveolar space (15). To avoid artificial cell dissemination during tumour dissection, each pathologist individually observed at least three tumour specimen sections. The dissemination of invasive adenocarcinoma through the airway cavity is shown in **Figures 2A–C**.

### Postoperative follow-up

Follow-up was conducted at the 3rd month after surgery and every 6 months thereafter. The follow-up data came mainly from the patient’s re-examination in the thoracic surgery clinic of our hospital and the follow-up centre of our hospital. For patients who were re-examined in local medical and health institutions, their follow-up data and examination data were collected through e-mail and telephone. Recurrence-free survival (RFS) was defined as the time from surgery to the first event(tumour recurrence or death from any cause) or the last follow-up. Overall survival (OS) was defined as the time from surgery to death or last follow-up. All recurrences were confirmed by clinical, radiologic, and pathologic assessment and were classified as local recurrence and distant recurrence. Local recurrence was defined as recurrence in the staple line,the ipsilateral hilar or mediastinal lymph node, ipsilateral pleura and ipsilateral lobes. Distant recurrence was defined as recurrence in the the contralateral hilar or mediastinal lymph node, contralateral lobes,contralateral pleura and other organs.

### Statistical methods

SPSS v26.0 (IBM, Armonk, NY, USA) was used for statistical analysis. Based on general clinical data, a multivariate logistic regression model built used to perform 1:1 matching between the experimental group and the control group according to the principle of nearest matching (matching tolerance: 0.02), the matching factors include Age, Sex, Smoking history, Histologic pattern, Vascular invasion, Pleural invasion, Lymphatic invasion, Pathologic stage, Lymph nodes resected. Pre-PSM clinical data were compared between groups by the χ^2^-test, and PSM clinical data were compared by the paired-sample t-test. Kaplan–Meier was used to assess DFS and OS. The Cox proportional-hazards regression model was used to evaluate the independent influencing factors of DFS and OS. All *p*-values are base on two-tailed statistical analysis, and *p* < 0.05 was statistically significant.

## Results

### Baseline characteristics of patients

A total of 3198 patients with Stage I lung adenocarcinoma underwent lobectomy between January 1, 2013, and January 1, 2016. STAS was observed in 992(31.0%) of all the patient.593 patients were enrolled in the study. The median age of onset was 62.0 (range 27.0-79.0) years, and the median follow-up time was 75.0 (range 11.0-99.0) months. The selection of adjuvant chemotherapy patients is determined jointly by the thoracic surgeon and oncologist who are treating the patient at the time. A total of 233 patients received postoperative ACT; they had a median age of onset of 61.0 (range 27.0-78.0) years and a median follow-up time of 75.0 (28.0-99.0) months. A total of 360 patients did not receive postoperative ACT; they had a median age of onset of 62.0 (30.0-79.0) years and a median follow-up time of 77.0 (11.0-99.0) months. Patients in the ACT group received 2-4 cycles of platinum-containing dual-drug chemotherapy after surgery, and there were no chemotherapy-related deaths.

In the whole sample, the ACT group and the non-ACT group had significant differences in vascular invasion (*p* = 0.047), visceral pleural invasion (*p* < 0.001, and clinical stage (*p <*0.001). After we grouped all patients by PSM (matching tolerance: 0.02), we obtained a total of 406 patients (203 cases in the ACT group, defined as the experimental group; 203 cases in the non-ACT group, defined as the control group). The baseline characteristics of the two groups were well matched, with no significant differences (**Table 1**).

**Table 1:** Variables of the two groups of the patients before PSM and afer PSM.

Variable	Before PSM			After PSM		
	Without ACT	With ACT	P-value	Without ACT	With ACT	P-value
Overall patients	360 (60.7)	233 (39.3)		203 (50.0)	203 (50.0)	
Age (years)			0.503			0.519
≤ 65	264 (41.1)	164 (27.7)		134 (33.0)	140 (34.5)	
>65	116 (19.6)	69 (11.6)		69 (17.0)	63 (15.5)	
Sex			0.983			0.678
Male	178 (30.0)	155 (19.4)		104 (25.6)	100 (24.6)	
Female	182 (30.7)	118 (19.9)		99 (24.4)	103 (25.4)	
Smoking History			0.761			0.667
Former/current	128 (21.6)	80 (13.5)		69 (17.0)	73 (18.0)	
Never	232 (39.1)	153 (25.8)		134 (33.0)	130 (32.0)	
Histologic pattern			0.177			0.374
Lepidic	6 (1.0)	2 (.03)		2 (0.5)	2 (0.5)	
Acinar	226 (38.1)	127 (21.4)		115 (28.3)	119 (29.3)	
Papillary	44 (7.4)	31 (5.2)		21 (5.2)	28 (6.9)	
Micropapillary	21 (3.5)	15 (2.5)		15 (3.7)	11 (2.7)	
Solid	63 (10.6)	58 (9.8)		50 (12.3)	43 (10.6)	
Vascular invasion			0.047			0.387
Absent	292 (49.2)	173 (29.2)		152 (37.4)	159 (39.2)	
Present	68 (11.5)	60 (10.1)		51 (12.6)	44 (10.8)	
Pleural invasion			< 0.001			0.139
Absent	284 (47.9)	122 (20.6)		127 (31.3)	118 (29.1)	
Present	76 (12.8)	111 (18.7)		76 (18.7)	85 (20.9)	
Lymphatic invasion			0.305			0.647
Absent	279 (47.0)	172 (29.0)		159 (39.2)	155 (38.2)	
Present	81 (13.7)	61 (10.3)		44 (10.8)	48 (11.8)	
Pathologic stage			< 0.001			0.100
StageIA1	18 (3.0)	0 (0)		0 (0)	0 (0)	
StageIA2	102 (17.2)	11 (1.9)		12 (3.0)	11 (2.7)	
StageIA3	91 (15.3)	50 (8.4)		52 (12.8)	50 (12.3)	
StageIB	149 (25.1)	172 (29.0)		139 (34.2)	142 (35.0)	
Lymph nodes resected			0.607			0.347
≤ 17	187 (31.5)	173 (29.2)		98 (24.1)	107 (26.4)	
>17	116 (19.6)	117 (19.7)		105 (25.9	96 (23.6)	

### RFS rates of the PSM groups

The median RFS time of the whole after PSM groups was 74.0 (11.0-99.0) months, the 3-year RFS rate was 87.4% (95%CI, 84.3% to 90.7%), and the 5-year RFS was 77.1% (95%CI, 73.1% to 81.3%). The median RFS of the experimental group was 75.0 (14.0-99.0) months, its 3-year RFS rate was 92.1% (95%CI, 88.5% to 95.9%), and its 5-year RFS rate was 83.3% (95%CI, 78.3% to 88.5%). The median RFS of the control group was 72.0 (11.0-99.0) months, its 3-year RFS rate was 82.8% (95%CI, 77.7% to 88.1%), and its 5-year RFS rate was 70.9% (95%CI, 65.0% to 77.5%).

**Figure 3:**
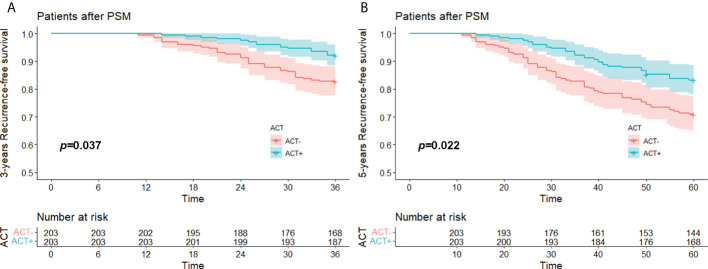
**(A)** 3 years-RFS and **(B)** 5 years-RFS of stage I (STAS^+^) patient with/without ACT after PSM. RFS, recurrence free survival; STAS, spread through air space; ACT, adjuvat chemotherapy; PSM, propensity-score matching.

Kaplan–Meier survival analysis showed that the difference in 3-year RFS between the experimental group and the control group was statistically significant (*p =* 0.037). The 5-year RFS difference between the experimental group and the control group was statistically significant (*p* = 0.022). The 3-year and 5-year RFS rates of the experimental group were both better than those of the control group (**Figures 3A, B**). A total of 34 patients relapsed within 5 years in the experimental group, 14 with local recurrence (1 in the staple line,6 in the ipsilateral hilar or mediastinal lymph node, 3 in the ipsilateral pleura and 3 in the ipsilateral lobes). and 20 with distant recurrence (10 in the the contralateral hilar or mediastinal lymph node, 6 in the contralateral lobes, 1 in the contralateral pleura and 3 in the other organs); a total of 59 patients relapsed within 5 years in the control group, 20 with local recurrence (1 in the staple line, 9 in the ipsilateral hilar or mediastinal lymph node, 2 in the ipsilateral pleura and 8 in the ipsilateral lobes) and 29 with distant recurrence (12 in the the contralateral hilar or mediastinal lymph node, 13 in the contralateral lobes, 1 in the contralateral pleura and 3 in the other organs).

The result of univariate analysis is showed in **Table 2**. Multivariate analysis removed two variables. Multivariate analysis by the Cox proportional-hazards regression model showed that vascular invasion (hazard ratio (HR): 4.399, 95% confidence interval (CI): 2.907-6.659; *p* < 0.001), visceral pleural invasion (HR: 2.056, 95% CI: 1.213-3.484; *p* = 0.007), and not receiving ACT (HR: 1.810, 95% CI: 1.184-2.766; *p* = 0.006) were independent influencing factors of RFS. Stage I STAS-positive adenocarcinoma patients who had vascular invasion or visceral pleural invasion or who did not receive postoperative ACT were more likely to relapse (**Table 3**).

**Table 2:** Cox proportional-hazards regression model for univariate analysis in the PSM.

		HR	95% CI	*P-*value
Recurrence-free Survival				
Sex	Male vs. Female	1.006	0.670-1.511	0.976
Smoking history	Current vs. never	0.867	0,491-1.645	0.308
Age (years)	≤65 vs. >65	0.820	0.538-1.251	0.357
Histologc pattern				0.932
Lepidic	Lepidic vs. solid	0.000	0.000-9.434E+200	0.963
Acinar	Acinar vs. solid	0.857	0.524-1.403	0.540
Papillary	Papillary vs. solid	1.105	0.560-2.182	0.773
Micropapillary	Micropapillary vs. solid	0.910	0.371-2.236	0.838
Solid	Solid vs.solid	1.000		
Vascular invasion	Present vs. absent	3.949	2.627-5.935	< 0.001
Pleural invasion	Present vs. absent	1.602	1.067-2.406	0.023
Lymphatic invasion	Present vs. absent	1.430	0.914-2.238	0.117
Pathologic stage				0.540
StageIA2	Stage IA2 vs. Stage IB	0.527	0.166-1.674	0.277
StageIA3	Stage IA3 vs. Stage IB	0.921	0.573-1.478	0.732
StageIB	Stage IB vs. Stage IB	1.000		
Lymph nodes	≤ 17 vs. >17	1.103	0.734-1.657	0.637
Adjuvant therapy	Absent vs. present	1.913	1.254-2.918	0.003
Overall Survival				
Sex	Male vs. Female	0.965	0.612-1.522	0.878
Smoking history	Current vs. never	0.967	0,691-1.845	0.408
Age (years)	≤ 65 vs. >65	0.784	0.490-1.254	0.310
Histologicpattern				0.944
Lepidic	Lepidic vs. solid	0.000	0.000-1.220E+229	0.968
Acinar	Acinar vs. solid	0.854	0.496-1.471	0.569
Papillary	Papillary vs. solid	1.060	0.493-2.279	0.882
Micropapillary	Micropapillary vs. solid	0.731	0.249-2.149	0.569
Solid	Solid vs.solid	1.000		
Vascular invasion	Present vs. absent	4.521	2.862-7.141	< 0.001
Pleural invasion	Present vs. absent	1.737	1.101 -2.741	0.018
Lymphatic invasion	Present vs. absent	1.711	1.052-2.784	0.031
Pathologic stage				0.526
StageIA2	StageIA2 vs. Stage IB	0.632	0.198-2.020	0.439
StageIA3	StageIA3 vs. Stage IB	0.773	0.443-1.349	0.365
StageIB	Stage IB vs. Stage IB	1.000		
Lymph nodes	≤ 17 vs. >17	1.097	0.695-1.731	0.691
Adjuvant therapy	Absent vs. present	1.778	1.111-2.844	0.016

**Table 3:** Cox proportional-hazards regression model for multivariate analysis in the PSM.

		HR	95% CI	*P-*value
Recurrence-free Survival				
Sex	Male vs. Female	1.223	0.802-1.867	0.349
Smoking history	Current vs. never	0.961	0.499-1.745	0.291
Age (years)	≤65 vs. >65	0.847	0.553-1.298	0.447
Vascular invasion	Present vs. absent	4.399	2.907-6.659	< 0.001
Pleural invasion	Present vs. absent	2.056	1.213-3.484	0.007
Lymphatic invasion	Present vs. absent	1.298	0.824-2.044	0.261
Pathologic stage				0.262
StageIA2	Stage IA2 vs. Stage IB	0.599	0.213-2.440	0.599
StageIA3	Stage IA3 vs. Stage IB	1.520	0.830-2.782	0.175
StageIB	Stage IB vs. Stage IB	1.000		
Adjuvant therapy	Absent vs. present	1.810	1.184-2.766	0.006
Overall Survival				
Sex	Male vs. Female	1.152	0.717-1.849	0.558
Smoking history	Current vs. never	0.867	0.641-1.945	0.508
Age (years)	≤ 65 vs. >65	0.299	0.483-1.250	0.777
Vascular invasion	Present vs. absent	5.014	3.154-7.969	< 0.001
Pleural invasion	Present vs. absent	2.086	1.162 -3.743	0.014
Lymphatic invasion	Present vs. absent	1.711	1.052-2.784	0.045
Pathologic stage				0.652
StageIA2	StageIA2 vs. Stage IB	0.861	0.249-2.980	0.813
StageIA3	StageIA3 vs. Stage IB	1.326	0.659-2.667	0.429
StageIB	Stage IB vs. Stage IB	1.000		
Adjuvant therapy	Absent vs. present	1.675	1.043-2.689	0.033

### OS rates of the PSM groups

The median OS time of the whole after PSM group was 74.8 (20.0-99.0) months, the 3-year OS rate was 97.3% (95%CI,95.4% to 98.7%), and the 5-year OS rate was 81.8% (95%CI,78.1% to 85.6%). The median OS of the experimental group was 75.0 (21.0-99.0) months, its 3-year OS was 98.5% (95%CI,96.9% to 100.0%), and its 5-year OS was 86.2% (95%CI,81.6% to 91.1%). The median OS in the control group was 74.0 (20.0-99.0) months, its 3-year OS rate was 96.1% (95%CI,92.8% to 98.4%), and its 5-year 0S rate was 77.3% (95%CI,71.8% to 83.3%).

**Figure 4:**
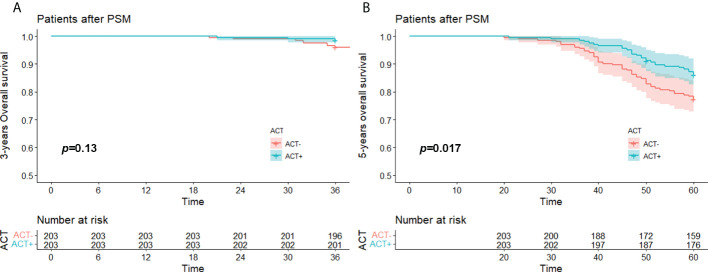
**(A)** 3 years-OS and **(B)** 5 years-OS of stage I (STAS^+^) patient with/without ACT after PSM. OS, over survival; STAS, spread through air space; ACT, adjuvat chemotherapy; PSM, propensity-score matching.

Kaplan–Meier survival analysis showed that there was no significant difference in 3-year OS between the experimental group and the control group (*p* = 0.13), so the experimental group did not have a survival advantage. However, the difference in 5-year OS between the experimental group and the control group was statistically significant (*p* = 0.017), so the 5-year survival rate of the experimental group was better than that of the control group (**Figures 4A, B**).

The result of univariate analysis is showed in **Table 2**. Multivariate analysis showed that stage I STAS-positive adenocarcinoma patients with lymphatic vessel invasion (HR: 1.711, 95% CI: 1.052-2.784; *p* = 0.045), vascular invasion (HR: 5.014, 95% CI: 3.154-7.969; *p* < 0.001), visceral pleural invasion (HR: 2.086, 95% CI: 1.162-3.743; *p* = 0.014), and not receiving ACT (HR: 1.675, 95% CI: 1.043-2.689; *p* = 0.033) brought significant survival disadvantages (**Table 3**).

### IA3 subgroup analysis

**Figure 5:**
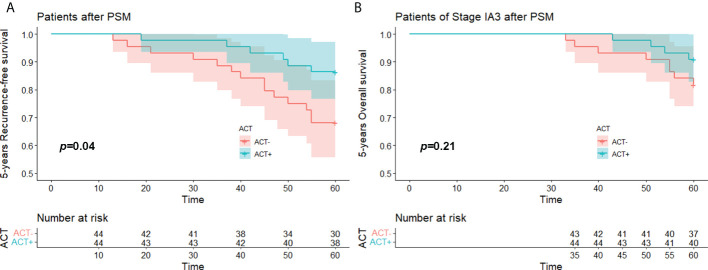
**(A)** 5 years-RFS and **(B)** 5 years-OS of stage IA3(STAS^+^) patient with/without ACT after PSM. OS, over survival; STAS, spread through air space; ACT, adjuvat chemotherapy; PSM, propensity-score matching.

We performed PSM on all 141 enrolled stage IA3 patients. The matched study group included 44 patients in the experimental group and 44 patients in the control group. The 5-year RFS of the experimental group was better than that of the control group (*p* = 0.040), but the 5-year OS did not show a significant difference (*p* = 0.21) (**Figures 5A, B**).

## Discussion

A growing number of studies have shown that lung adenocarcinoma with STAS is associated with a higher risk of postoperative recurrence and a lower survival rate in patients with lung adenocarcinoma (9, 16–19). Related studies have also reported a better prognosis of stage I lung adenocarcinoma patients with STAS who underwent lobectomy than those who underwent segmentectomy (11, 20). However, there are few reports on whether stage I lung adenocarcinoma patients with STAS can benefit from ACT after lobectomy. To our knowledge, this is the first PSM study on the whether patients with stage I lung adenocarcinoma (STAS^+^) can benefit from postoperative ACT.

Chen (20) confirmed that STAS is a clear prognostic factor for patients with stage IA and IB lung adenocarcinoma and proposed that the patients with stage IA STAS^+^ lung adenocarcinoma had similar RFS and OS as patients with stage IB STAS^–^ lung adenocarcinoma. Here, we further confirmed that ACT was an independent factor affecting the long-term survival of patients with stage I (STAS^+^) lung adenocarcinoma who underwent lobectomy, and more specifically, stage I (STAS^+^) lung adenocarcinoma patients who did not receive ACT after lobectomy had a higher risk of recurrence (HR: 1.810, 95% CI: 1.184-2.766; *p* = 0.006) and death (HR: 1.675, 95% CI: 1.043-2.689; *p* = 0.033).

The need of patients with stage I lung adenocarcinoma to undergo ACT, especially those with stage IA lung adenocarcinoma who have undergone lobectomy, has been continuously questioned. In our study, due to the small number of stage IA1 and IA2 patients included in the total sample of 593, we only analysed a subgroup of 141 stage IA3 patients. We separately matched these 141 patients with PSM and obtained a post-PSM study that included 44 pairs of patients. In this study groups, the 5-year RFS of patients who received ACT was significantly better than that of patients who did not receive ACT (*p* = 0.040). Although the 5-year OS survival curve showed a certain differentiation trend, the difference was not statistically significant (*p* = 0.21). This may have been due to the small number of stage IA3 patients enrolled in the study and the small number of deaths within 5 years. The 5-year RFS of the patients already showed a difference, so with a longer follow-up time, the OS of the patients might also show differences.

In recent years, a growing number of studies have explored the predictors of postoperative benefit from ACT in patients with stage I lung cancer (21–24). A multicentre study by Japanese scholars confirmed that ACT after lobectomy in stage I lung adenocarcinoma patients with visceral pleural invasion, lymphatic vessel invasion, and vascular invasion could not only prolong the RFS of the patients but also increase the OS. In our study, lymphatic vessel invasion (HR: 1.711, 95% CI: 1.052-2.784; *p* = 0.045), vascular invasion (HR: 5.014, 95% CI: 3.154-7.969; *p* < 0.001), and visceral pleural invasion (HR: 2.086, 95% CI: 1.162-3.743; *p* = 0.014) were independent influencing factors of long-term survival after lobectomy for stage I STAS^+^ lung adenocarcinoma. STAS is another invasion method besides the above three. Wang et al. (24) confirmed that ACT can benefit the postoperative survival of stage I lung adenocarcinoma with micropapillae as the main component. the presence of STAS is positively correlated with adenocarcinoma subtypes with a predominantly micropapillary or solid component (25, 26). Although 59.5%(353/593) of the patients in our study with acinar as main component,but 82.6%(490/593)of the patients had micropapillae or/and solid components,this ratio is similar to that in Lee’s study (26). Therefore, our study further confirmed that ACT could benefit the survival of patients with stage I (STAS^+^) regardless of the presence of micropapillary/solid components.

Our study has certain limitations. First, although we performed PSM on the study subjects in this retrospective study, there may still be a certain selection bias. Second, our limited follow-up time may be the reason why stage IA3 patients who received ACT did not show any survival benefit. Third, it is controversial whether any part of the free-floating tumour cell clusters identified by pathologists as STAS was artificially created *in vitro*. Most notably, tumour cell clusters may be spread by the knife surface during processing of the specimen, known as “spread through a knife surface (STAKS)” (27). Although we observed at least three tumour sections of each specimen under a microscope, we still inevitably included some STAKS patients rather than real STAS patients in the study. Due to the small number of cases, we did not perform subgroup analysis of the morphology of STAS or the prognosis of ACT. In addition, the decision-making about ACT and the choice of chemotherapy regimen were based on the subjective preference of the attending physician rather than a randomized choice.

In summary, ACT is a favourable prognostic factor for patients with stage IB(STAS^+^) lung adenocarcinoma. For stage IA3 patients, ACT improves the RFS, but it brings no advantage to OS. A larger, longer-term clinical observational study is needed to explore the role of ACT in the treatment of patients with STAS^+^ IA3 lung adenocarcinoma.

## Data availability statement

The raw data supporting the conclusions of this article will be made available by the authors, without undue reservation.

## Ethics statement

The studies involving human participants were reviewed and approved by the committee of the Forth Hospital of Hebei Medical University (No.2021ky103). The ethics committee waived the requirement of written informed consent for participation.

## Author contributions

SX: Conceptualization, Data curation, Writing - original draft. YH: Data curation. HD: Formal analysis, Writing - review & Editing. SW: Data curation, Formal analysis. GL: Investigation, Methodology. QL: Conceptualization, funding acquisition, Writing - review & Editing. All authors contributed to the article and approved the submitted version.

## Funding

This work was supported by Medical science research project of Hebei province (Grant No.20201076), The programme of the government funding Clinical Excellence of Hebei province (2019 Grant No.139).The programme of the government funding Clinical Excellence of Hebei province (2022 Grant No.180).

## Acknowledgment

We thank American Journal Experts for language editorial assistance, Yueping Liu M.D. for pathological image assistance.

## Conflict of interest

The authors declare that the research was conducted in the absence of any commercial or financial relationships that could be construed as a potential conflict of interest.

## Publisher’s note

All claims expressed in this article are solely those of the authors and do not necessarily represent those of their affiliated organizations, or those of the publisher, the editors and the reviewers. Any product that may be evaluated in this article, or claim that may be made by its manufacturer, is not guaranteed or endorsed by the publisher.
